# Neuropsychological Abnormalities Associated with Alcohol Dependence During Long-Term Rehabilitation Treatment of German Inpatients

**DOI:** 10.3390/brainsci14111160

**Published:** 2024-11-20

**Authors:** Josef Rabl, Dieter Geyer, Dario Kroll, Fabrizio Schifano, Norbert Scherbaum

**Affiliations:** 1 Department of Psychiatry and Psychotherapy, LVR-University Hospital Essen, Faculty of Medicine, University of Duisburg-Essen, Virchowstr. 174, 45147 Essen, Germany; josef.rabl@stud.uni-due.de; 2 Johannesbad Kliniken Fredeburg GmbH, Zu den drei Buchen 1, 57392 Schmallenberg, Germany; 3 MIDI-Insitute, Faculty of Applied Social Sciences, Technische Hochschule Köln, Gustav-Heinemann-Ufer 54, 50968 Köln, Germany; dariokroll@posteo.de; 4 Psychopharmacology, Drug Misuse and Novel Psychoactive Substances Research Unit, School of Life and Medical Sciences, University of Hertfordshire, College Lane Campus, Hatfield AL10 9AB, UK; f.schifano@herts.ac.uk

**Keywords:** alcohol use disorder, craving, stop–signal–reaction-task, impulsivity, attentional bias, longitudinal design, rehabilitation treatment

## Abstract

Background: Alcohol dependence is associated with several neuropsychological abnormalities, such as increased impulsivity or attentional bias towards drug-related stimuli. However, it is debated whether these abnormalities are on the decline after long-term abstinence from alcohol. Inpatient rehabilitation treatment enables the longitudinal investigation of such variables during a long, largely secured, period of abstinence. Methods: This study involved alcohol-dependent patients consecutively admitted for a duration of 14–26 weeks to an inpatient rehabilitation treatment center located in a hospital specializing in substance use disorders. Craving and impulsivity were assessed with the means of two questionnaires (e.g., OCDS-G and BIS-11); conversely, attentional bias and problems with inhibition were measured with the help of two computer-based experiments (e.g., dot–probe task and stop–signal–reaction task). Investigations were conducted at entry, after 6 weeks, and during the last two weeks of the inpatient treatment. Results: A total of 130 patients with alcohol dependence (mean age 43.3 years; 78.5% male) completed the first, *N* = 102 the second, and *N* = 83 the final assessment. Over the whole period of inpatient treatment, there was a significant decrease in patients’ scores for both craving (t(83) = 7.8, *p* < 0.001) and impulsivity (t(82) = −3.75, *p* < 0.001, t(82) = 4.4, *p* < 0.001). However, there were no significant changes regarding attentional bias (t(82) = 0.16, *p* = 0.494) and inhibitory control (t(76) = 0.04, *p* = 0.482) scores. Conclusions: Neuropsychological abnormalities associated with alcohol dependence might persist even after a long abstinence period. The decrease in both craving and impulsivity levels may be explained by the protected, alcohol-free, hospital environment; however, patients’ risk of post-discharge relapse may remain high, as the basic neurobiological mechanisms of alcohol dependence may persist for long periods, and possibly for more than 3–6 months.

## 1. Introduction

Alcohol dependence is associated with a range of psychological issues, including reduced functionality of working memory [1,2], reduced performance in response–inhibition tasks [3], increased levels of both craving [4] and impulsivity [5,6,7], and a shift in attention towards alcohol-related cues (e.g., attentional bias) [8,9,10]. It remains to be seen whether these neuropsychological issues are either the result of current and regular alcohol intake or a possibly acquired trait that may persist even after a long period of abstinence from alcohol, hence possibly representing a relapse risk factor.

These neuropsychological issues are arguably related to neurobiological changes associated with the neurotoxic effects of alcohol [11]. It remains to be seen if long-term alcohol abstinence during rehabilitation treatment may be associated with levels of brain regeneration. Indeed, an increase in formerly altered grey and white matter volumes in alcohol-dependent patients due to prolonged abstinence of 7.5 months was demonstrated [12], with improvements possibly varying among the different brain regions [13]. When compared to healthy controls after 7 months of abstinence, volume deficits in alcohol substance use disorder (SUD) patients were, however, still present in all areas, apart from the prefrontal cortex [14]. In addition to the effects of alcohol abstinence, psychotherapeutic treatment, such as cognitive behavioral therapy [15] during the rehabilitation period, might positively influence abnormalities such as craving [16] and impulsivity [17].

There is a large body of research focusing on neuropsychological abnormalities in SUD patients compared with healthy controls. These studies often present a range of methodological problems, including different methodological approaches; varying, and at times small, sample sizes; different experimental setups; and other shortcomings [18]. According to a systematic review [18] focusing on 95 studies, merely 5 were evaluated as possessing a “strong quality” and 23 a “good quality”. Attentional bias regarding AUD patients was assessed in *N* = 25 studies, with mixed results. Indeed, 9 studies reported an attentional bias in AUD patients in comparison to healthy controls, 14 studies did not show any difference between groups, and 3 identified an avoidance bias in AUD patients. Given these cross-sectional studies’ mixed results, longitudinal studies assessing the course of neuropsychological abnormalities in SUD inpatients may provide a better understanding of the relevance of neuropsychological abnormalities in persons with SUD. However, only 13 studies from the above-mentioned review [18] were conducted with the help of a within-subject design, and none of them investigated the attentional bias of SUD patients. Similar limitations apply to other neuropsychological measures, such as inhibitory control and impulsivity [19,20]. Regarding alcohol craving, a recent review summarized a range of interesting studies, but all focused on patients attending a short-term, treatment-assisted withdrawal program and not a long-term rehabilitation treatment center [21]. Results from observation periods longer than a few weeks could be explained by country-specific circumstances. Indeed, research findings relating to alcohol detoxification treatment in both the UK and the USA referred to periods of 3–14 days (for outpatient and inpatient treatment [22,23]), with many patients not having been engaged in post-detox alcohol treatment [24].

In Germany, long-term inpatient rehabilitation treatment is widely available and accessible to persons with SUD as it is paid for either by pension funds or, in some cases, by statutory health insurance. The duration of inpatient rehabilitation treatment ranges from 14 weeks up to half a year, depending on the patient’s main substance use disorder. Prior to admission to a rehabilitation hospital, patients usually undergo an inpatient detoxification treatment, lasting up to 21 days. Long-term rehabilitation treatment consists of a broad variety of treatment approaches, including weekly individual psychotherapy sessions focusing, e.g., on craving management, better containment of negative emotions, and reducing impulsivity; group psychotherapy sessions, held on several occasions per week; occupational assessment and therapy; sports therapy; career and social counseling; and the facilitation of post-discharge connection with local self-help groups. During inpatient treatment, patients’ alcohol abstinence is confirmed through regular unannounced breathalyzer and urine sample tests (e.g., the identification of ethyl glucuronide). A recent paper highlighted differences between the treatment of alcohol dependence in the United States and Germany [25].

Prospective studies seem to be crucial for a better understanding of possible changes over time in neuropsychological abnormalities in SUD patients. The aim of the current investigation was to assess whether highly specialized, long-term, and intensive inpatient treatment resulted in substantial improvements in those neuropsychological issues possibly relating to the dependent use of alcohol in treatment-compliant inpatients. In particular, we investigated whether neuropsychological functions such as attentional bias, inhibitory control, impulsivity, and alcohol craving showed any modifications over the course of the long-term residential treatment. The following hypotheses were tested:

1. The patients’ alcohol craving, as measured by the OCDS-5 score, will significantly decline over the course of the treatment.
2. The patients’ attentional bias (dot–probe score) will significantly decline over the course of the treatment.
3. The patients’ impulsivity (BIS11 and UPPS scores) will significantly decline over the course of the treatment.
4. The patients’ inhibitory control (SSRT score) will significantly increase over the course of the treatment.

## 2. Materials and Methods

A prospective, longitudinal, observation study was conducted in the Johannesbad Fachklinik Fredeburg (FKF) clinic in Western Germany. The clinic provides rehabilitation treatment for inpatients with different substance use disorders; its typical treatment duration varies between 14 and 26 weeks.

### 2.1. Participants

The minimum sample size of the study was computed using G*Power [26,27]. The parameters were set whilst estimating a treatment medium effect of d = 0.3 on the neuropsychological abnormalities, an alpha error of 0.05, and a power of 0.80. This resulted in a minimal sample size of *N* = 71. Participants were recruited between the 4 July 2022 and the 15 April 2023. Data collection was finalized on 11 July 2023. Inclusion criteria were <65 years old and alcohol dependence according to ICD-10; exclusion criteria were insufficient knowledge of the German language, with specific reference to a lack of understanding of either the experiment-related instructions or how to fill in the questionnaires. Furthermore, patients with a current diagnosis of schizophrenia spectrum disorder (F2 according to ICD-10) were excluded. In fact, the affected patients were typically too ill to join such a study, and their treatment differed from the remaining patients, with possibly additional medications and/or even transfer to a psychiatric hospital needed. Due to the need to carry out a range of PC-based experiments, epilepsy was here excluded as well. Possible other psychiatric comorbidities were not considered a reason for participant exclusion. Eligible patients were asked to participate in the study; those who expressed an interest were informed about the study both verbally and in writing and signed the consent form prior to formally being included in the study.

### 2.2. Materials

Alcohol craving, impulsivity, inhibitory control, and attentional bias towards alcohol-related cues during long-term inpatient treatment were here considered as the variables of interest. All participants took part in the routine treatment, which included repeated satisfactory confirmation of their alcohol abstinence. Exploratory variables such as time of abstinence before treatment, days spent in treatment until the third experiment, age, and possible comorbidities were assessed. Data on comorbidities regarding other SUDs or possible other mental disorders were provided by the clinical files.

### 2.3. Procedure

The study experiments were conducted at three points in time: during the participants’ first week of treatment; after six weeks of treatment; and during the last two weeks of treatment. Experiments were presented on a 19-inch screen with a 4:3 aspect ratio. Participants used two keys on a QWERTZ keyboard to complete tasks and a mouse to answer the questionnaires. The software Inquisit Lab 6 [28] supported the reaction time experiments as well as the analysis of the questionnaires.

### 2.4. Alcohol Craving

Due to both its ease of use and proven reliability [29,30], the short German version of the Obsessive Compulsive Drinking Scale (OCDS-G) was here administered to measure the patients’ cravings. The reliability was satisfying at all three points of measurement (Cronbach’s α at t1 = 0.88, at t2 = 0.86, and at t3 = 0.79). To prevent the following experiments from impacting the participants’ cravings, this was the first assessment administered to participants.

### 2.5. Attentional Bias

The alcohol dot–probe experiment [31,32] was the next assessment carried out. Here, a fixation cross was presented for 500 ms, after which, two images appeared alongside each other for a duration of 1000 ms; they showed either random objects/probes or simultaneous non-alcoholic and alcoholic beverages images. Stimuli were always shown side by side and paired, meaning that relevant probes consisted of an alcoholic beverage next to a non-alcoholic one and control probes showed a pair of random objects. Following the stimuli, a probe (a white “X”) was presented in the same position as one of the pictures displayed shortly before, either until the response of the participant was provided, or for the duration of 1000 ms in case of no response. Participants were instructed to press one of two assigned keys regarding the probes’ position on the screen as precisely and as fast as possible. The experiment measured the participants’ attentional bias towards alcohol by comparing the reaction time in alcohol-congruent (e.g., the probe that followed the alcohol stimuli) versus non-congruent (e.g., the probe following non-alcoholic stimuli) trials. The calculated difference over all trials, e.g., a faster reaction time in alcohol-congruent trials, measured in milliseconds, indicated an attentional bias. However, if the result was negative, meaning that the task was worked on more slowly in alcohol-congruent trials than regarding alcohol-incongruent trials, such values were also considered in the longitudinal analyses. Due to their North American origin, the original stimuli, referring to the pictures of alcoholic beverages used by the group of Miller and Fillmore, were here modified, and pictures of German alcoholic beverages were provided instead.

### 2.6. Impulsivity

Impulsivity was assessed using the German versions of both the Urgency, (lack of) Premeditation, (lack of) Perseverance, and Sensation Seeking (UPPS) [31,33,34] and the Barratt Impulsiveness Scale 11 (BIS-11) [35,36,37] questionnaires. Both questionnaires were presented by using the PC and Inquisit Lab 6. The summarized scores of both questionnaires’ scales were automatically generated by Inquisit and used for the statistical analyses. Reliability was excellent for the UPPS-G scale (Cronbach’s α at t1 = 0.91, at t2 = 0.91, and at t3 = 0.92) and good regarding the BIS-11 scale (Cronbach’s α at t1 = 0.85, at t2 = 0.88, and at t3 = 0.88).

### 2.7. Inhibitory Control

To assess the patients’ inhibitory control, the stop–signal–paradigm measuring inhibitory control was here used [38,39]. A fixation circle, where an arrow appeared after a short duration, randomly pointing left or right, was presented. Participants were instructed to press the assigned keys on the keyboard as precisely and as fast as possible to indicate the arrows’ direction. After the arrow appeared, a short beep was randomly played via the computer’s speakers. The beep’s delay started at 250 ms and was automatically adjusted by the software in 50 ms increments up or down, depending on the participant’s performance. Participants were instructed to inhibit their reaction when this beep (e.g., the stop signal) appeared. The beep’s volume was set to match each participant’s hearing ability. If, consistent with the consensus guide to stop–signal tasks [39], a violation of the test’s paradigm was identified, the dataset of the participant was excluded from the calculations. The resulting stop–signal reaction times were computed in milliseconds by Inquisit and used for the statistical analyses.

### 2.8. Demographic Data

The clinic provided sociodemographic and clinical data routinely collected for descriptive analysis and further explorative analyses. For demographic and diagnostic data, see Table 1 and Table 2.

### 2.9. Statistical Analyses

Statistical analyses were computed via R version 4.2.2 [40] using the graphical user interface RStudio, version 2023.06.0, build 421 [41]. Participants’ cravings, attentional bias, impulsivity, and inhibitory control were analyzed for significant differences. Therefore, we computed Repeated Measure ANOVAs for each variable over the course of the patients’ treatment. Mauchly’s Test for Sphericity was computed to assess if the ANOVAs’ requirement of sphericity was given. The Greenhouse–Geisser adjustment was utilized to account for violations of sphericity. For further analyses, we computed paired-sample t-tests, utilizing the Bonferroni correction, leading to a level of significance threshold of *p* < 0.016 instead of *p* < 0.05. Cohen’s d values were calculated for effect sizes. Linear models were utilized to assess the influence of exploratory variables.

## 3. Results

A total of *N* = 172 patients were invited to join the study and *N* = 152 agreed. Out of these 152 patients, 18 patients were, however, excluded from participation due to a range of reasons, including the need for urgent medical treatment, the presence of secondary information relating to diagnoses that were consistent with the exclusion criteria, refusing further participation over the course of the rehabilitation treatment itself, the presence of cognitive limitations preventing proper understanding of the tasks, and having relapsed prior to the first experiment. In addition, three patients did not attend the experiment appointments and, hence, were excluded from the study, and, finally, a computer issue caused the total loss of data relating to a single participant.

In total, 130 patients completed the first experiment, 102 completed the second, and 83 completed the third experiment. Reasons for drop-outs from the study included premature termination of the rehabilitation treatment (*N* = 34), revocation of participation (*N* = 4), relapse into substance use (*N* = 2), and organizational issues, e.g., absence of research assistant due to illness (*N* = 7). The treatment of two participants was prolonged after the third experiment; hence, there was a fourth experiment for two participants that replaced the third experiment. Participants who completed all three experiments were included in the Repeated Measures ANOVA (*N* = 80 regarding OCDS-5, attentional bias, BIS-11, and UPPS; *N* = 74 regarding SSRT).

Out of 130 participants, 102 were males; the mean age of participants was 43.3 years (SD = 11.09; range: 18–64). Regarding education, 20.8% of participants had completed high school, 61.5% had completed vocational training or middle school, and 4.6% had a university diploma (see Table 1). Of all participants, 76.2% presented with a diagnosis of tobacco dependence (ICD-10: F17.2), 48.5% were dependent on a further substance (excluding nicotine), and 31.6% were dependent on three or more substances (see Table 1).

Psychiatric comorbidities were identified in 50.8% of the sample (see Table 2). The mean number of days spent in the clinic until the third experiment was 103.4 (SD = 27.48). The mean number of days of abstinence before admission to the rehabilitation clinic was 34.4 (SD = 44.10) and the mean duration of treatment (including patients who terminated treatment prematurely) was 85.8 days (SD 40.88).

### 3.1. Main Results

We computed the Shapiro–Wilk normality test for the variables: normal distribution of the data was given only in case of the UPPS score (W = 0.99, *p* = 0.33), but not regarding the OCDS-5 Craving Scale (W = 0.89, *p* < 0.001), attentional bias (W = 0.92, *p* < 0.001), BIS-11 (W = 0.98, *p* < 0.05), and SSRT (W = 0.96, *p* < 0.001).

The Repeated Measures ANOVAs showed a significant change in the variables OCDS-5 (F([2], [158]) = 50.50, *p* < 0.001, η^2^g = 0.20), BIS-11 (F([2], [158]) = 8.52, *p* < 0.001, η^2^g = 0.01), and UPPS (F([2], [158]) = 18.27, *p* < 0.001, η^2^g = 0.02). No significant change was found regarding attentional bias (F([2], [158]) = 1.72, *p* = 0.181, η^2^g = 0.01) and SSRT (F([2], [146]) = 0.04, *p* = 0.957, η^2^g = 0.00).

Mauchly’s Test for Sphericity showed a violation of the assumption of sphericity concerning all variables: OCDS-5 Craving Scale (W = 0.54, *p* < 0.001), attentional bias (W = 0.83, *p* < 0.001), BIS-11 (W = 0.91, *p* < 0.05), UPPS (W = 0.73, *p* < 0.001), and SSRT (W = 0.67, *p* < 0.001). Hence, all calculations were corrected with the Greenhouse–Geisser correction to account for the lack of sphericity, with the result that all significant effects proved to be robust to the correction (see Table 3).

### 3.2. Further Analyses

Mean values and standard deviations of the main variables, together with the effect size in case of a significant change in the variable over time, are shown in Table 4. As the numbers of participants completing experiments 2 and 3 (see above) were different, the mean value is given here for each time and each comparison. To test changes over time, for each variable of interest, one-tailed paired t-tests were used.

A significant decline in craving levels over time, as measured by the OCDS-G, was here identified. Indeed, the subsample that completed experiments 1 and 2 scored significantly higher at the beginning of the treatment on the OCDS-G scale than 6 weeks afterwards (t(101) = 7.07, *p* < 0.001), with the decline possibly being interpreted as a medium effect (Cohen’s d = 0.70). In comparing craving levels at six weeks vs. the end of treatment, a significant (t(80) = 3.5, *p* < 0.001), but small (Cohen’s d = 0.39), effect was observed. Comparisons between data at the time of experiment 1 vs. experiment 3 confirmed a significant reduction in alcohol cravings (t-test, t(83) = 7.8, *p* < 0.001), albeit with a medium effect (Cohen’s d = 0.69).

Attentional bias, as measured via the alcohol dot–probe task, declined over time from experiment 1 to experiment 2, albeit with no significant effect (t(101) = 2.06, *p* = 0.020). Similarly, the comparisons (t2 to t3: t(79) = −1.57, *p* = 0.939; t1 to t3: t(82) = 0.16, *p* = 0.494) did not show any significant reductions over time. Impulsivity, as measured via self-reports, showed a decrease over the course of the whole treatment duration. The BIS-11 impulsivity scale did not show a significant reduction from experiment 1 to experiment 2 (e.g., t(101) = 1.70, *p* = 0.0455). A medium effect (Cohen’s d = 0.38) was observed when comparing the BIS-11 scores at experiment 2 vs. experiment 3 (t(79) = 3.43, *p* < 0.001). In addition, there was a decrease in the BIS-11 score over the whole treatment duration (t(82) = −3.75, *p* < 0.001, Cohen’s d = 0.41). Similarly, the UPPS-G scores showed a statistically significant decrease over time for all three calculations; a small effect (Cohen’s d = 0.26) was observed for the decrease from experiment 1 to experiment 2 (t(100) = 2.61, *p* < 0.01) and from experiment 2 to experiment 3 (t(78) = 4.29, *p* < 0.001). Conversely, a medium effect (Cohen’s d = 0.48) was observed when comparing the decrease in levels from experiment 1 to experiment 3 (t(82) = 4.4, *p* < 0.001). Measurements of inhibitory control via the stop–signal task did not show a significant reduction in reaction times over the course of the treatment. All three one-tailed paired t-tests (t12(96) = 0.03, *p* = 0.486; t23(75) = −0.27, *p* = 0.608; t13(76) = 0.04, *p* = 0.482) did not show any significant decrease in reaction time levels when comparing the three measurements’ results.

The effects on the variables of interest, at the time point of the first experiment, of age, days spent in abstinence before treatment, and number of comorbidities were calculated using linear regression models. The former days of abstinence reported by participants were associated with decreasing effects on both cravings (F(1, 128) = 7.766, t = −2.787, *p* < 0.05, η^2^ = 0.0572) and impulsivity, as measured with the UPPS-G (F(1, 127) = 6.536, t = −2.557, *p* < 0.05, η^2^ = 0.0489). There was a significant influence of the number of substances being misused on a number of parameters, leading to an increase in cravings (F(1, 128) = 8.88, t = 2.980, *p* < 0.05, η^2^ = 0.0648), BIS11 scores (F(1, 128) = 21.86, t = 4.676, *p* < 0.001, η^2^ = 0.146), and UPPS-G scores (F(1, 127) = 19.09, t = 4.369 *p* < 0.001, η^2^ = 0.13). The number of psychiatric comorbidities other than SUD showed a significant influence on BIS11 scores (F(1, 128) = 17.06, *p* = < 0.001, η^2^ = 0.117). Patient age was associated with significant decreasing effects on BIS11 scores (F(1, 128) = 11.75, t = −2.796, *p* < 0.001, η^2^ = 0.084) and UPPS-G scores (F(1, 127) = 11.08, t = −2.56, *p* = 0.001, η^2^ = 0.080) and an increase in reaction times for the stop-signal-task (F(1, 124) = 14.8, t = 3.866, *p* < 0.001, η^2^ = 0.106).

## 4. Discussion

To the best of our knowledge, this paper represents the only longitudinal investigation focusing on changes in neuropsychological abnormalities during long-term inpatient rehabilitation treatment for alcohol dependence. A range of heterogeneous results regarding the different variables were here identified, including, most notably, a decrease over time in both self-reported cravings and impulsivity. These modifications occurred in parallel with a small effect size reduction in attentional bias, but only between the first and second experiments, with a lack of any significant modifications over the course of the treatment of inhibitory control scores.

The self-reported alcohol cravings showed a consistent significant reduction over time, up to the end of treatment (e.g., comparison of measurements 2 and 3), backing the assumption that prolonged abstinence and treatment would have positive effects on patients’ cravings. However, although the current participants presented with a diagnosis of alcohol dependence, the overall baseline score for craving was low; this finding is consistent with the intensity of craving being linked to perceived substance availability levels, and rehabilitation clinics are, indeed, substance-free environments [8,42]. The clinic location was over 1.4 km away from the nearest facility selling alcoholic beverages, and participants were not allowed to drive during treatment. Another possible explanation for the decrease in cravings is that control is regained after the improved function of the dorsolateral prefrontal cortex (DLPFC) resulting from prolonged abstinence [43]. It has to be noted, however, that during the course of the treatment, all participants were exposed to cognitive behavioral therapy sessions, where craving coping strategies were discussed.

The patients’ self-reported impulsivity levels reduced significantly during the course of the rehabilitation treatment. This may have increased patients’ ability to make more rational choices associated with better control over alcohol intake behavior, with this finding having possibly been the result of the intensive therapeutic intervention provided to all patients [44]. At a neurobiological level, the prolonged abstinence and the positive effects of the regeneration of crucial areas in the brain that are responsible for self-control could account as well for the decreasing levels observed regarding impulsivity [45,46].

There were no significant changes in patients’ attentional bias levels towards alcohol-related cues between the first and final experiments. Indeed, the stop–signal task reaction times did not show here any improvements over time. One could have expected an attentional bias reduction associated with the rehabilitation treatment provided, in itself facilitated by the regeneration of those brain areas responsible for cognitive control such as the dorsal anterior cingulate cortex (dACC), DLPFC, and dorsal parietal cortex (DPC) [14,43]. However, it might take longer than a few weeks/months for the brain to regenerate in those areas after mostly decades of alcohol abuse [14,43]. The relatively low mean and the large standard deviation levels of the attentional bias here identified may suggest that there are many patients with substantially positive, as well as many patients with substantially negative, attentional bias levels. Only positive values in the alcohol dot–probe-paradigm are usually interpreted as an attentional bias; however, a strong negative value (e.g., a slow reaction time in alcohol-congruent trials) might be elicited by the patients’ emotional valence towards alcohol stimuli and their attempt to control the emerging desire or rising negative emotions. Hence, it is here suggested that both positive and negative relevant deviations of attentional bias-related measures might be interpreted as neuropsychological abnormalities. Reduced reaction times might be a consequence of a focus on the addictive substance, but prolonged reaction times might indicate high levels of cognitive loads associated with the presentation of the addictive substance, e.g., due to rising negative emotions or memories of relapse.

Those explicit measures based on self-reports of patients regarding craving and impulsivity showed improvement, whereas the computer-based implicit measures did not. In the secure and substance-free environment of the rehabilitation clinic, alcohol-dependent participants may well have experienced lower levels of impulsivity and craving. Conversely, other underlying neuropsychological abnormalities may conceivably persist, even after lengthy inpatient admission, which lasted here for an average of about 3 months. These considerations may somehow explain the high levels of early relapse events just after the completion of rehabilitation treatment [47]. Indeed, a recent study reported a relapse rate of 40.5% within the first three months after discharge from an inpatient rehabilitation treatment center [48]. The persistence of high levels of attentional bias and a lack of inhibitory control may well interfere with the goal of abstinence when patients return to their homes, where, usually, the availability of alcohol may suddenly and drastically increase, alongside all the hassles of daily life.

The discrepancy between the results of the implicit, computerized experiments versus those of the self-report questionnaires might also be explained by a social desirability bias [49]. It can be assumed that patients in a rehabilitation treatment center would like to please both the therapeutic staff and also themselves, thus reporting a treatment-related subjective improvement. This bias might be further increased by the improved social interaction levels of participants during inpatient treatment in a rehabilitation clinic, as alcohol dependence is often accompanied by social isolation. In contrast, the results of computer-based experiments are less at risk of being influenced by the social desirability bias. To reduce the impact of the social desirability bias, the participants were assured by the investigator that the treatment team would not be informed about the results of the questionnaires and tests.

Whilst abstinence was overall assured here due to the clinic’s regulations and random inspections, it is not fully guaranteed that participants maintained full alcohol sobriety during their whole inpatient treatment. Furthermore, measuring the levels of implicit neuropsychological parameters in a secure and controlled clinical environment may limit their generalizability in real-world scenarios. Furthermore, craving and impulsivity measurements relied on self-reports, with the associated risk of response bias. The investigated sample consisted of patients suffering not only from alcohol dependence but often from other SUDs as well. This may indeed reflect the reality of both inpatient and outpatient drug addiction clinics. An exclusion of patients with comorbid substance-related disorders would have led to a very low sample size. AUD patients commonly present with comorbid mental disorders, especially affective disorders [50,51]. Comorbid mental disorders could influence the results of neuropsychological tests, e.g., depression was shown to be associated with reduced cognitive performance [52]. This could call into question the validity and generalizability of our measures. However, we made a conscious decision not to exclude patients with comorbidities (except schizophrenia spectrum disorders), as we wanted to assess a representative sample of German AUD inpatients, including common comorbidities. This decision should have hence increased the validity and generalizability of the results of this study. Whilst the experiments were here carried out with high standards, the use of more advanced technology, such as eye-tracking, was not here made available.

## 5. Conclusions

Current, albeit conflicting, findings relating to the study of explicit and implicit measures could contribute to a better understanding of the high alcohol relapse rates observed even after an extensive (e.g., weeks or months) duration of inpatient rehabilitation treatment. The present findings showed that whilst self-reported measures of craving and impulsivity significantly reduced over time, implicit measures of attentional bias and inhibitory control did not in parallel significantly change. Therefore, even after intensive rehabilitation treatment, the relapse risk is high, and follow-up treatment is necessary. Future studies should elucidate whether the implicit abnormalities should be interpreted as persisting traits or whether they may be reduced after a longer period (e.g., more than 3–6 months) of alcohol abstinence.

## Figures and Tables

**Table 1:** Demographic data of the sample, *N* = 130.

	Frequency (%)	M (SD)	Min/Max
Age		43.31 (11.09)	18/64
Female	28 (21.53%)		
Male	102 (78.46%)		
Educational background			
No degree	6 (4.61%)		
Basic degree	11 (8.46%)		
Secondary school (10th class or vocational training)	80 (61.53%)		
High school	27 (20.76)		
University diploma	6 (4.61)		
Number of SUD diagnoses *		2.27 (1.002)	1/6
1	26 (20.0%)		
2	63 (48.5%)		
3 or more	41 (31.5%)		
Number of psychiatric diagnoses		0.68 (0.828)	0/4
0	64 (49.2%)		
1	50 (38.5%)		
2 or more	16 (12.3%)		

Demographic characteristics of the sample, frequencies, and percentages of co-morbidities regarding psychiatric diagnoses in the patients; tobacco addiction was not included in the calculation. * We excluded nicotine dependence from the SUD diagnoses as tobacco smoking was not prohibited in the clinic and, hence, patients were not checked on abstinence from nicotine uptake.

**Table 2:** Comorbid substance-related disorders and other mental disorders of the sample (frequencies and percentages).

	Frequency	%
Substance-related diagnoses		
Tobacco dependence (F17.2)	99	76.2
Opioid dependence (F11.2)	1	0.8
Cannabinoid dependence (F12.2)	26	20.0
Dependence on sedatives/hypnotics (F13.2)	3	2.3
Cocaine dependence (F14.2)	11	8.5
Stimulant dependence (F15.2)	13	10.0
Other psychiatric diagnoses		
Mood [affective] disorders (F3)	43	33.0
Neurotic, stress-related, and somatoform disorders (F4)	23	17.6
Behavioral syndromes associated with physiological disturbances and physical factors (F5)	1	0.8
Disorders of adult personality and behavior (F6)	10	10.7
Behavioral and emotional disorders with onset usually occurring in childhood and adolescence (F9)	6	4.6

**Table 3:** Repeated Measures ANOVAs with Greenhouse–Geisser correction.

Variable	df_Num_	df_Den_	Epsilon	F	*p*	η^2^g
OCDS-5	1.37	108.50	0.69	50.50	0.000	0.20
Attentional Bias	1.72	135.96	0.86	1.73	0.186	0.01
BIS-11	1.84	145.74	0.92	8.53	0.000	0.01
UPPS	1.58	125.04	0.79	18.28	0.000	0.02
SSRT	1.51	109.92	0.75	0.04	0.919	0.00

dfNum = degrees of freedom numerator, dfDen = degrees of freedom denominator, Epsilon = Greenhouse–Geisser multiplier for degrees of freedom. *p*-values and degrees of freedom in the table incorporate this correction. η^2^g = generalized eta-squared.

**Table 4:** Means, standard deviations, and effect sizes of statistically significant differences over time.

	t1 M (SD)	t2 M (SD)	*t3* M (SD)	Cohen’s d t1 to t2 t2 to t3 t1 to t3
Alcohol Craving OCDS-5				
Score at t1	5.34 (3.71)	-	-	*d*_12_ = 0.69
included in comparison to t2	5.25 (3.62)	2.93 (2.49)	2.25 (2.14)	*d*_23_ = 0.39
included in comparison to t3	5.4 (3.66)	2.94 (2.36)	2.25 (2.14)	*d*_13_ = 0.85
Attentional Bias Alcohol Dot–Probe Paradigm				
deviation in milliseconds at t1	1.46 (26.32)	-	-	
included in comparison to t2	3.72 (27.46)	−3.99 (24.75)	0.88 (19.79)	*-*
included in comparison to t3	1.23 (26.85)	−4.56 (26.1)	0.74 (19.46)	*-*
Impulsivity BIS-11				
Score at t1	67.49 (10.75)	-	-	
included in comparison to t2	67.44 (10.73)	66.35 (10.61)	65.42 (10.82)	-
included in comparison to t3	67.76 (10.92)	67.44 (10.17)	65.02 (10.86)	*d*_13_ = 0.41
UPPS-G				
Score at t1	109.81 (16.6)	-	-	*d*_12_ = 0.26
included in comparison to t2	109.59 (16.11)	107.8 (14.91)	105.81 (15.81)	*d*_23_ = 0.48
included in comparison to t3	110.48 (15.74)	109.05 (14.39)	105.48 (16,01)	*d*_13_ = 0.48
Inhibitory Control Stop–Signal Task (Integration method, milliseconds)				
Score at t1	218.14 (57.24)	-	-	-
included in comparison to t2	218.04 (54.71)	217.8 (51.13)	219.94 (32.71)	-
included in comparison to t3	221.71 (57.41)	218.38 (52.48)	221.46 (35.59)	-

Mean, standard deviations, and effect sizes of significant differences over time, if given. t1 = experiment 1 at beginning of treatment; t2 = experiment 2 six weeks into treatment; t3 = within the second-to-last or last week of treatment. M = mean; SD = standard deviation; d = Cohen’s d, only given if the difference was statistically significant. Included in comparison to t2 = mean of the subsample that was compared with experiment 2 at t1/t3; for t2, the mean of the subsample that was compared with t1 is given. Included in comparison to t3 = mean of the subsample that was compared with experiment 3 at t1/t3; for t2, the mean of the subsample that was compared with t1 is given. N_OCDS-5_ at t1 = 130, N_OCDS-5_ included in comparison t1 with t2 = 102, N_OCDS-5_ included in comparison t2 with t3 = 81, N_OCDS-5_ included in comparison t1 with t3 = 84.

## Data Availability

Complete raw data are not publicly available, but de-identified data could be made available upon reasonable request from the corresponding author.
